# Editorial: Stakeholders' perspectives on assessment and improvement of quality in early childhood education and care: a world-wide kaleidoscope

**DOI:** 10.3389/fpsyg.2024.1448246

**Published:** 2024-07-22

**Authors:** Linda Joan Harrison, Antonia Elisabeth Enikoe Baumeister, Hui Li

**Affiliations:** ^1^School of Education, Faculty of Arts, Macquarie University, Sydney, NSW, Australia; ^2^Institute of Psychology, Chemnitz University of Technology, Chemnitz, Germany; ^3^Department of Early Childhood Education, Faculty of Education and Human Development, The Education University of Hong Kong, Tai Po, Hong Kong SAR, China

**Keywords:** early childhood education and care (ECEC), educational quality, assessment, stakeholders, social validity, quality improvement, curriculum frameworks, quality

Aligned with the 2030 United Nations Sustainable Development Goals (2015) Goal 4, the key aims of early childhood education and care (ECEC) are to offer children from all social backgrounds a good start in their lives, to support parenting as well as families' workforce participation, and, thereby, to sustainably strengthen the national economy over current and future generations. High-quality ECEC has been shown to improve child outcomes and be a buffer against developmental risk factors. For these reasons, governments, ECEC providers, and researchers are increasingly focusing on the frameworks and systems that underpin quality and the measures that assess quality. Meanwhile, policy-related evidence shows that the aims and benefits of high-quality ECEC can only be reached when all stakeholders' needs are acknowledged and sufficiently met.

This Frontiers Topic aimed to promote research as a multidisciplinary endeavor that would derive internationally significant conclusions about the opportunities and obstacles in assessing and delivering quality ECEC at national and local levels. We suggest that diverse, wide-ranging stakeholder input would generate innovative methods for assessing and improving quality that keep pace with today's rapidly changing society. To this end, we broadly define stakeholders to include government and non-government regulatory agencies, ECEC service providers, teachers and educators (or caregivers), families, communities, and children.

Our call for expressions of interest in this Frontiers Research Topic attracted responses from authors, associate editors, and reviewers located across 6 continents and 14 countries. We received 22 manuscripts; of which, 2 were withdrawn, 16 were accepted, and 4 were rejected. Three Frontiers journals were involved in the review/publication process:

- Frontiers in Psychology, with its section Educational Psychology,

- Frontiers in Education, with the sections Educational Psychology and Leadership in Education, and
- Frontiers in Public Health, with its section on Public Health Policy.

We are grateful to many experts who supported the editorial process and/or reviewed each of the articles carefully, including focusing specifically on their structural, conceptual, and linguistic levels. Their generous input has contributed to the high quality and readability of the published articles, which include conceptual analyses, policy and practice reviews, a brief report, and original research. Collectively, these 16 articles illustrate the systemic interlinking of multiple steps toward engaging stakeholders in conceptualizing and assessing quality, quality improvement, and professionalization. The studies feature a variety of research methodologies, many of which illustrate the creativity of scientists in the application of innovative methods, for example, to respectfully gather the views and insights of First Nations communities, as well as of children and young people.

Inspired by Bronfenbrenner and Morris's (2006) bioecological model of human development, we propose a “spiral model” of ECEC research and policy development. In Figure 1, we seek to visually capture the development of processes and sequences from the micro-system steps of “stakeholder needs analysis” through conceptualization and the “definition of framework guidelines” for quality ECEC and the “development of implementation methods” to assess quality. Beyond these are macro-systems and macro-time processes of “evaluation of implementation,” leading to the “derivation of further action strategies” and “re-analyses and reforms.” The x-axis of the model represents the time dimension (chronosystem) as micro-, meso-, and macro-time. The y-axis represents the different levels at which the systemic processes of quality assessment and improvement in ECCE take place from micro-systems (e.g., childcare groups, centers, and communities) to macro-systems (e.g., national frameworks and cross-cultural comparisons).

**Figure 1:**
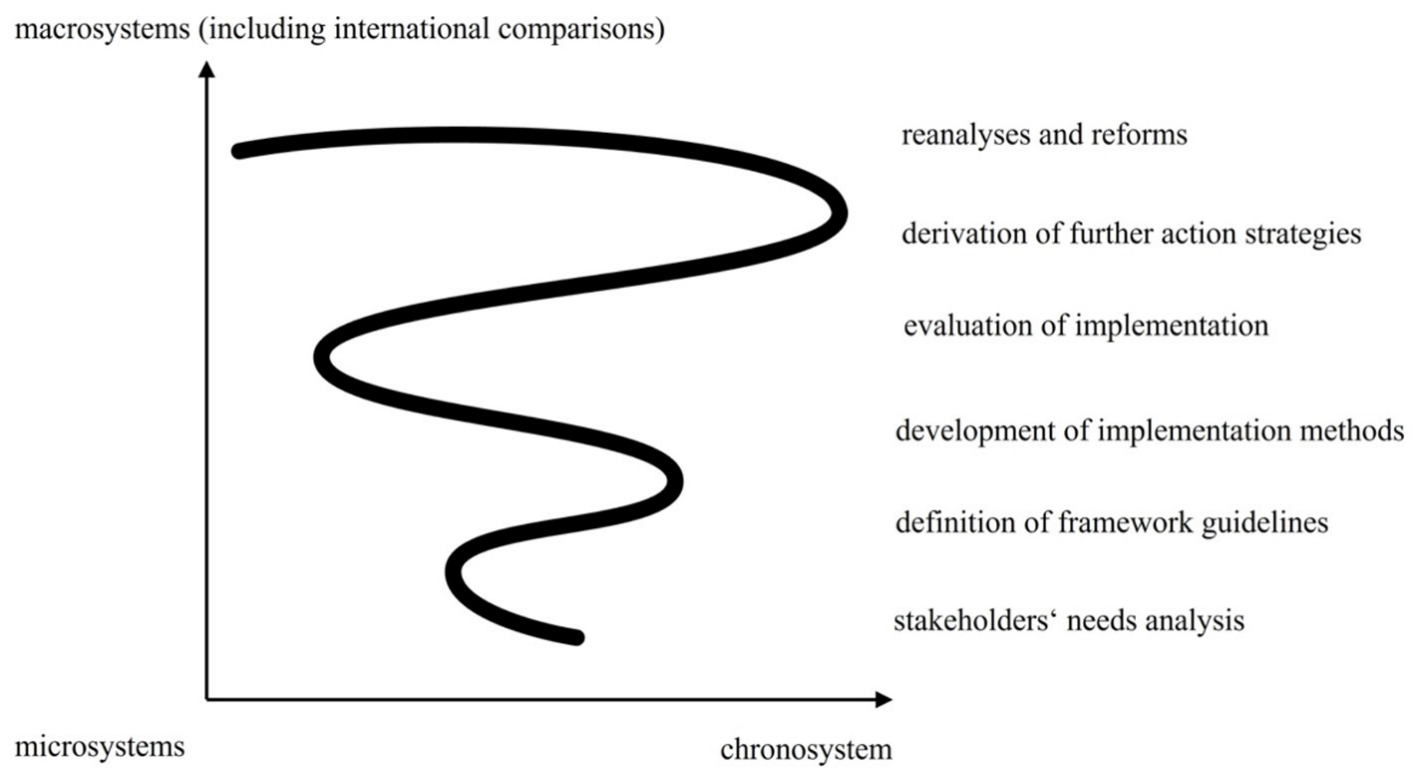
The spiral model of quality assessment and improvement in ECEC.

Our “spiral model of quality assessment and improvement in ECEC” was inspired by the responses of authors, reviewers, and editors to the Research Topic invitation to explore and discuss models for gathering the perspectives of multiple stakeholders and considering the significance of stakeholder views for conceptualizing, assessing, and improving quality in ECEC. The 16 accepted articles illustrate three different aspects of the spiral model:

(1) engaging stakeholders in ECEC research and policy development through comprehensive and creative approaches to needs analysis;
(2) conceptualizing and assessing quality in ECEC through definitions, implementation methods, and evaluation; and
(3) professionalization and quality improvement in ECEC through action strategies, re-analyses, and proposals for reforms.

Four studies illustrate different national and local approaches to *Engaging Stakeholders in ECEC Research and Policy Development*. Addressing macro-system ECEC reform, Hadley et al. described the principles and theoretical underpinnings of an inclusive, nationwide stakeholder engagement strategy and its application in a mixed-method sequential design that aimed to contemporize and update Australia's national frameworks for ECEC and school-age care services. Modes of engagement included indirect online surveys for service providers, teachers, educators, other professionals, and families; direct interviews, Delphi discussions, and focus groups; written submissions from individuals and organizations; and educator-facilitated conversations and drawings by children and young people. Skattebol et al. proposed and tested the approachability, acceptability, affordability, accessibility, and appropriateness (5 As) model of engagement to address the critical challenge of access and uptake of ECEC services by families experiencing social and economic adversities, using an iterative Delphi method with 23 high-level, experienced stakeholders.

Cartmel et al. highlighted the role of children and young people as stakeholders in policy reform, describing the design of creative, educator-facilitated methodologies to engage and support them in expressing their ideas. Interested educators were provided with briefings and written information on gathering informed consent, using dialogic drawing, talking circles, and visual elicitation methods, and diarizing their reflections on the images and ideas generated by participating children. Adamson and Skattebol applied a targeted approach to engage stakeholders with specialized, local knowledge of ECEC services in remote areas of Australia with significant populations of First Nations peoples and children under 5 years of age. Their approach aimed to understand and address the low ECEC attendance rates (16%) through input from both Indigenous and non-Indigenous community members.

Seven articles discuss various aspects of *Conceptualizing and Assessment of Quality in ECEC* and present diverse perspectives and approaches to defining, quantifying, and analyzing quality in center and home-based ECEC services. Pianta and Hofkens summarized evidence from a large number of studies conducted in preschools and kindergartens in the United States and 12 other countries using the Classroom Assessment Scoring System (CLASS) indicators of teacher-child emotional support, instructional support, and classroom management. Cohrssen et al. drew on the Australian ECEC context to consider differing stakeholder priorities for quality, as demonstrated by assessment outcomes based on the National Quality Standard (NQS), regulatory indicators, family perceptions, and research-based conceptualizations. Phillips and Boyd applied Bronfenbrenner's ecological theory to explore the intersection of national standards, leadership, governance, relationships, and personal qualities in an in-depth study of ECEC services that had achieved the highest NQS rating of exceeding. Baumeister et al. focused on the quality of home-based childcare services in an evaluation of a participatory procedure for assessing quality by providers and parents, *the Educational and Parenting Test for Home-Based Childcare*. Their findings from a German sample of non-relative caregivers, parents, and experts in ECEC pedagogy show how the acceptance of quality assessment can be achieved among stakeholders through opportunities to participate in the process of quality development. Participatory examination of “what” quality is, and “how” and “when” it is achieved is further explored by Grieshaber and Hunkin in an ethnographic study conducted with Australian educators and pre-service teachers. Responses tap the tangible and intangible aspects of quality, such as what quality “feels like” and how it is created. The final articles in this section draw on *NQS Assessment and Rating* (A&R) data made available by the *Australian Children's Education and Care Quality Authority* (www.acecqa.gov.au). Davis et al. used Leximancer semantic mapping to examine changes in educators' documented *Quality Improvement Plans* over two rounds of *NQS* A&R. The findings showed that greater emphasis was given to management, leadership, and professional development in centers that had improved from working toward to exceeding NQS. Char et al. analysis of systems-level predictors of quality in home-based ECEC services provides further evidence of the critical role of governance in supporting quality outcomes.

Five articles address *Professionalization and Quality Improvement* issues by exploring various stakeholder engagement strategies. Embacher and Smidt's survey of Austrian preschool teachers investigated the relationship between professional competencies (e.g., work engagement) and the quality of observed teacher–child interactions, assessed using the individualized *CLASS* (inCLASS) version. Irvine et al. used a case study methodology to gather insights from ECEC providers, leaders, and educators. Their study focused on experiences of the NQS A&R process, particularly working in centers that had improved their NQS ratings. Siraj et al. implemented a randomized controlled trial (RCT) methodology to test the “Leadership for Learning” professional development program. This program aimed to improve the quality and development outcomes of preschool-aged children in Australia. Using quantitative and qualitative methods, Buchner et al. evaluated the effectiveness of intercultural “anti-bias” training and reflection sessions among a group of German educators. Boese et al. tested the effectiveness of professional language support training for educators working with bilingual children in Germany using an intervention vs. historical control group comparison.

Overall, the collection of articles in this Research Topic is crucial for governments, ECEC providers, teachers, educators, and the scientific community. This Research Topic emphasizes the role of stakeholders in research that aims to measure, understand, achieve, and improve the quality of ECEC services. It also highlights the critical importance of professional learning in fostering quality practices and supporting children's learning, development, and wellbeing. These scholarly articles contemporize best practices and propose new solutions for conceptualizing, measuring, and enhancing ECEC quality.

## Author contributions

LH: Conceptualization, Writing – review & editing. AB: Conceptualization, Visualization, Writing – original draft. HL: Conceptualization, Writing – review & editing.
